# Multiple Sympathetic Affections Caused by the Presence of Intestinal Worms

**Published:** 1881-01

**Authors:** 


MULTIPLE SYMPATHETIC AFFECTIONS CAUSED BY THE PRESENCE OF INTESTINAL WORMS.—Dr. Guermonprez (*Journal des sciences med. de Lille*, July, 1880), reports this curious case: A girl eleven years old, without morbid antecedents, and lacking all hereditary taint, had occasionally passed some longround worms, (*ascaris lumbricoides*), but in less number than many other children living in the same locality. Her father noticed that her intelligence began to weaken and that she showed peculiarities of character. She would tear her clothes, run away from home, or grow morose, irritable and perverse. Her memory began to fail her. At irregular intervals, and without appreciable cause, these psychical disturbances became even more marked. Her nights would then be spent in restless agitation. This lasted about a month. The restlessness was at that time constant; when about, she must walk; if stopped, she stamped her feet, kicked with irregular movements, gave no answers. When seated, her limbs were in perpetual motion. There were moments of apparent calmness, but also decided exascerbations. Hearing was lost and vision became impaired. In addition, speech forsook her and her face was distorted by grimaces. Frequently she cried; at other times she appeared to be viciously inclined, pursuing her sister with a knife. Again, she would be the victim of actual hallucinations. The bodily functions were regularly performed, but she had grown somewhat thinner. Opium, chloral, bromide of potassium and baths all proved unavailing in the cure of her malady. At length a vermifuge was tried, and thirty-seven worms were passed. On the following day the nervous condition was visibly improved. In twelve days she passed about eighty worms, and as she got rid of them her agitation subsided and her intelligence returned. In two months she had completely recovered, and the cure has remained undisturbed for two years since that time.—*Gazette med de Paris.*

